# A retrospective cohort study of patients with stomach and liver cancers: the impact of comorbidity and ethnicity on cancer care and outcomes

**DOI:** 10.1186/1471-2407-14-821

**Published:** 2014-11-07

**Authors:** Diana Sarfati, Jason Gurney, James Stanley, Jonathan Koea

**Affiliations:** Department of Public Health, University of Otago Wellington, PO Box 7343, Wellington, 6242 New Zealand; University of Otago Wellington, PO Box 7343, Wellington, 6242 New Zealand; Waitemata District Health Board, Takapuna, Auckland, New Zealand

**Keywords:** Comorbidity, Ethnicity, Cancer, Hepatic neoplasms, Gastric neoplasms, Māori, New Zealand, Survival

## Abstract

**Background:**

Comorbidity has an adverse impact on cancer survival partly through its negative impact on receipt of curative treatment. Comorbidity is unevenly distributed within populations, with some ethnic and socioeconomic groups having considerably higher burden. The aim of this study was to investigate the inter-relationships between comorbidity, ethnicity, receipt of treatment, and cancer survival among patients with stomach and liver cancer in New Zealand.

**Methods:**

Using the New Zealand Cancer Registry, Māori patients diagnosed with stomach and liver cancers were identified (n = 269), and compared with a randomly selected group of non-Māori patients (n = 255). Clinical and outcome data were collected from medical records, and the administrative hospitalisation and mortality databases. Logistic and Cox regression modelling with multivariable adjustment were used to examine the impacts of ethnicity and comorbidity on receipt of treatment, and the impact of these variables on all-cause and cancer specific survival.

**Results:**

More than 70% of patients had died by two years post-diagnosis. As comorbidity burden increased among those with Stage I-III disease, the likelihood that the patient would receive curative surgery decreased (e.g. C3 Index score 6 vs 0, adjusted OR: 0.32, 95% CI 0.13-0.78) and risk of mortality increased (e.g. C3 Index score 6 vs 0, adjusted all-cause HR: 1.44, 95% CI 0.93-2.23). Receipt of curative surgery reduced this excess mortality, in some cases substantially; but the extent to which this occurred varied by level of comorbidity. Māori patients had somewhat higher levels of comorbidity (34% in highest comorbidity category compared with 23% for non-Māori) and poorer survival that was not explained by age, sex, site, stage, comorbidity or receipt of curative surgery (adjusted cancer-specific HR: 1.36, 95% CI 0.97-1.90; adjusted all-cause HR: 1.33, 95% CI 0.97-1.82). Access to healthcare factors accounted for 25-36% of this survival difference.

**Conclusions:**

Patients with comorbidity were substantially less likely to receive curative surgery and more likely to die than those without comorbidity. Receipt of curative surgery markedly reduced their excess mortality. Despite no discernible difference in likelihood of curative treatment receipt, Māori remained more likely to die than non-Māori even after adjusting for confounding and mediating variables.

**Electronic supplementary material:**

The online version of this article (doi:10.1186/1471-2407-14-821) contains supplementary material, which is available to authorized users.

## Background

Patients with cancer often carry the dual burden of the cancer itself and other co-existing chronic conditions. Comorbidity can have a substantial impact on the patient, their clinicians and the health services in general [1–5]. The role of comorbidity in the care and outcomes of cancer patients is complex with the inter-relationships between comorbidity, receipt of treatment and outcomes for cancers poorly understood. The impact of these factors in explaining disparities in cancer survival between patients in different ethnic or socioeconomic groups remains even less clear.

Comorbidity is known to have a negative impact on the likelihood of receiving curative treatment among cancer patients generally [6–14]. For example, a systematic review of studies analysing the use of chemotherapy among stage III colon cancer patients in the US reported that seven out of nine studies found comorbidity had a deleterious effect on chemotherapy receipt [15]. The magnitude of the effect was large with the odds ratios comparing the uptake of chemotherapy among patients with Charlson (global measure of comorbidity) scores of 2 or 3+ with those with scores of 0 ranging from 0.38-0.44 in one large study [15]. The impact of comorbidity on treatment for stomach and liver cancers specifically has not been established.

This issue is important because whilst it is clear that those with comorbidity have poorer cancer survival, it is not entirely clear the extent to which this occurs due to the direct effect of comorbidity or through its impact on treatment choice or effectiveness. Intuitively it is likely that both play a part. Few studies have investigated this issue, but those that do suggest that, among cancer patients, at least part of the excess mortality among those with comorbidity is due to lower receipt of definitive treatment [13, 14].

Comorbidity is common among cancer patients in general, but those in ethnic minority and lower socioeconomic groups frequently carry a greater burden of chronic disease than others [16–19]. These same groups often experience poorer cancer survival [19–22]. Comorbidity has been shown to be in part responsible for these disparities in cancer survival. For example, a study by Hill et al. [20] showed that a third of the disparity in colon cancer survival between Māori (Indigenous New Zealanders) and non-Māori New Zealanders was due to comorbidity. Studies in other countries have similarly found that disparities in cancer survival between indigenous and non-indigenous populations are, at least partially, explained by differences in levels of comorbidity [19, 23]. In the US, the evidence relating to the impact of comorbidities on ethnic/racial inequalities in outcomes is inconsistent. Several authors have found that comorbidity partially or completely explains such disparities [14, 24–29], while others have concluded that comorbidity may not be important in this regard [30–32].

This study aims to investigate the inter-relationships between comorbidity, receipt of treatment, ethnicity and cancer survival among a cohort of patients with liver and stomach cancers in New Zealand. Internationally, stomach and liver cancers are the third- and fourth leading causes of cancer deaths respectively [33].

The specific objectives of this study are to investigate: 1) the impact of comorbidity on receipt of curative treatment; 2) the impact of comorbidity on all-cause and cancer-specific survival, and the proportion of any excess mortality explained by lack of receipt of treatment and 3) the extent to which comorbidity and receipt of definitive treatment explains differences in survival between Māori and non-Māori New Zealanders with liver or stomach cancer.

## Methods

### Data sources

Incident cases of stomach (ICD-10-AM code: C16.×) and hepatocellular (C22.0) cancers diagnosed between 1 Jan 2006 and 31 Dec 2008 were identified from the New Zealand Cancer Registry (NZCR). These cancers were combined because there were insufficient numbers of either alone for this study, and there are similarities between these cancers in that both are associated with chronic infection, smoking and alcohol consumption, so are similarly related to comorbidity; both are treated by upper gastrointestinal surgeons and have surgery as their primary treatment modality, and both are associated with similarly poor survival.

New Zealand has mandatory reporting of all cancers (except non-melanoma skin cancer). Patients were eligible for inclusion if they a) were aged 25 years or older at diagnosis; b) were normally resident in New Zealand; c) had no previous diagnosis of the same primary cancer; and d) were diagnosed prior to their death. New Zealand is divided into two main Islands (North and South), with nearly 80% of the population (and specifically 90% of the Māori population) based in the North Island [34]. All eligible Māori patients (who make up 15% of the total population) residing in the North Island were included, along with an equal number of randomly-sampled non-Māori patients. The purpose of this was to ensure that the study had equal explanatory power for Māori and non-Maori. Ethnicity was classified on the basis of Cancer Registry data which uses an ever-Māori approach, where patients are classified as Māori if they have been identified as Māori on any previous health record. All other patients were classified as non-Māori.

Clinical data relating to each patient were extracted from all relevant public and private hospitals in the North Island by a trained oncology nurse. Data were recorded on a standardised study pro-forma, double-entered and any discrepancies resolved. Details of this process are available elsewhere [35]. Data were also collected from routine administrative hospital records (National Minimum Dataset) for the five years prior to diagnosis (for assessment of comorbidity), and the national mortality collection up to the end of 2010 (providing a minimum of two years of follow-up data for all patients). Approval for this study was granted by the New Zealand Multi-Region Ethics Committee (MEC/10/042/EXP), and permission for access to the data given by the Ministry of Health and relevant District Health Boards.

### Variables

Sex, age at diagnosis, and prioritised ethnicity (Māori or non-Māori) were determined from the Cancer Registry. Socioeconomic deprivation and urban/rural classification were determined using the domicile of residence data recorded on the Cancer Registry at time of diagnosis. Deprivation was measured using the NZDep index, a small-area based index calculated using aggregated census data based on residents’ socioeconomic characteristics (such as benefit receipt, earning under an income threshold, housing tenure, access to car or phone, etc.) [36]. Higher values of the NZDep index indicate greater deprivation.

The clinical notes review provided data on details of patient’s presentation (including a specified list of comorbid conditions present at the time of diagnosis), tumour characteristics (including tumour grade, and stage at diagnosis, classified according to the TNM classification system [37]), and receipt of treatment (including surgery, chemotherapy, radiotherapy and palliative care). Curative surgery was defined as surgical intervention among those with Stage I-III disease for whom the treatment intent was curative.

Comorbidity was measured in two ways. First, the 12 most common comorbid conditions identified in the notes review were included in the analysis. Conditions were treated individually, or as a categorised ‘count’ to assess the overall burden of comorbidity at diagnosis. Second (and separately), all conditions recorded in the administrative hospitalisation data in the five years prior to diagnosis were identified and used to calculate a C3 comorbidity index score for each patient [38]. The C3 index is a cancer-specific index of comorbidity based on the presence of 42 chronic conditions each of which is weighted to its impact on one-year non-cancer mortality in a cancer cohort [39]. These weights are then summed to arrive at a final comorbidity (C3 Index) score. For descriptive analysis of the study cohort, C3 Index scores were categorised into ‘0’ (C3 Index score < =0), ‘1’ (0 < score < =1), ‘2’ (1 < score < =2) and ‘3’ (score >2) Comorbid conditions that may have been complications of the primary disease or its treatment were only included if they were recorded prior to the date of diagnosis or index date of admission (specifically myocardial infarction, congestive heart failure, pulmonary embolism, anxiety/behavioural disorders, anaemias, hypertension and cardiac arrhythmias). In addition, conditions that may have been indicative of early malignancy were excluded; specifically liver disease and upper gastrointestinal ‘comorbidity’ were excluded when calculating the C3 index score for patients with liver and stomach cancers, respectively.

### Statistical analysis

Māori, non-Māori and total cohorts were compared for demographic and disease characteristics, patient comorbidity and receipt of definitive treatment. Because all Māori patients were included but only a subset of non-Māori patients, the estimates reported for the total cohort were weighted to the total eligible Māori and non-Māori stomach and liver cancer populations. When reporting estimates stratified by ethnicity, rates were age-standardised to the total New Zealand cancer population (2006–2008) using direct standardisation. These analyses were repeated restricted to those with stage I-III disease only. To assess the association between ethnicity and comorbidity, we fitted a linear regression model with the C3 score as the continuous outcome, in order to estimate a mean difference in C3 score adjusting for age as a continuous predictor using restricted cubic splines (see next paragraph). We assessed two-year all-cause and cancer-specific survival using a Kaplan-Meier approach.

Next, a series of multivariable models were fitted to explore the relationships between comorbidity, ethnicity, treatment and survival. In these models age was treated as a continuous variable, and modelled using restricted cubic splines with knots at the 5^th^, 50^th^, and 95^th^ percentiles. Comorbidity was treated in two ways in these models. When comorbidity was the primary independent variable the C3 index score was used, treated as a continuous variable and included using restricted cubic splines with knots at the 5^th^, 50^th^, and 95^th^ percentiles [40]. When comorbidity was being treated as a confounding or mediating variable, the C3 index was included in models (using splines as described above), alongside a continuous comorbidity count from the hospital notes review data.

To assess the extent to which comorbidity and ethnicity impacted on the receipt of definitive treatment, patients with Stage IV cancer were excluded from the analysis (since treatment for these patients is indicated for palliative purposes only). First, a logistic regression model was fitted examining the impact of comorbidity on receipt of definitive treatment. Age (modelled as continuous, with restricted cubic splines), sex (M/F), site (liver/stomach), deprivation (treated as a continuous linear predictor, in deciles), rurality (rural/non-rural) and ethnicity (Māori/non-Māori) were all treated as potential confounders because they are common causes of both comorbidity and receipt of treatment (see Additional file 1: Figure S1 for causal diagrams). Stage (categorised I, II, III) was fitted last because comorbidity may impact stage at diagnosis (although the direction and magnitude of this impact is unpredictable) [41, 42], and stage in turn has an impact on whether or not definitive treatment can be offered and so could be considered both a confounder and a mediator in this relationship.

Next, we assessed the impact of receipt of definitive treatment on all-cause and cancer-specific survival using Cox proportional hazards regression. For these analyses, age, sex, site, stage, ethnicity, deprivation, rurality and comorbidity (using both measures) were all considered confounders and included in the model (See Additional file 1: Figure S1 for causal diagrams).

We then assessed the impact of comorbidity (C3 Index scores) on survival, and the extent to which this was mediated by receipt of definitive treatment. For these analyses, age, sex, site, ethnicity, deprivation and rurality were considered confounders. Stage of disease was fitted next (for aforementioned reasons), followed by receipt of definitive treatment.

To assess whether ethnicity had an impact on receipt of definitive treatment, and the extent to which this association was mediated by comorbidity, a logistic regression model was fitted. For these models, age, sex and site were considered confounders and were added to the model first, followed by stage. Stage was considered a mediator because ethnicity is likely to impact on stage of diagnosis through factors such as uneven access to primary care services, which in turn impacts on treatment. We were interested in the impact of comorbidity once the effect of stage at diagnosis had been accounted for. Comorbidity was added to the model (using both the C3 index score and the count of the number of comorbidities) to estimate the extent to which the potential remaining effect of ethnicity on receipt of treatment was caused by differential levels of comorbidity between ethnic groups. Access to healthcare factors (deprivation and rurality) were added last as additional potential mediators in this association.

Finally, to assess the impact of ethnicity and comorbidity on (all-cause and cancer-specific) survival, Cox regression models were fitted following the same sequential model adjustment protocol as outlined above (with the addition of receipt of curative treatment). For all Cox regression models, individuals were censored at the end of follow up time. For cancer-specific analyses, patients were censored at their date of death if they died of non-cancer causes.

All analyses were carried out in SAS v9.2. Those models with exposures fit with restricted cubic spline variables were conducted using an add-in macro [40] which also produces solutions of the odds/hazard ratios at specified points in the exposure distribution.

## Results

There were 269 Māori patients and 255 non-Māori patients (total n = 524) included in this study. Patient characteristics are shown in Table 1. Patients with stomach cancer comprised 64% of the total cohort. Males formed the majority of the cohort (67% versus 33% female). The overall mean age was 64 years (SD = 15), with the Māori cohort having a younger mean age (Māori: 60 years, SD 14; Non-Māori: 68 years, SD 14; *p* <0.001). A substantial proportion of the cohort was recorded as having late-stage disease, with 43% diagnosed at Stage IV. There were 293 patients with stage I-III disease. The profile for stage of disease at diagnosis was similar for Māori and non-Māori. Māori were considerably more likely to reside in areas of high deprivation than non-Māori (age-adjusted proportion residing in NZDep deciles 9–10, Māori: 60%, non-Māori: 27%; *p* <0.001). Māori were also less likely to reside in an urban area compared to non-Māori (age-adjusted proportion residing in urban area, Māori: 65%, non-Māori 83%; *p* <0.001). More than 70% of all patients in both ethnic groups and for both stomach and liver cancers had died within two years of diagnosis (Table 2). Additional file 1: Table S1 shows patient characteristics for those diagnosed with Stage I to III disease only; with the distribution of characteristics being very similar as for the full cohort.

**Table 1:** **Characteristics of the study population: proportions by site sex, age, stage, deprivation, rurality and comorbidity**

			Māori			Non-Māori			
	Total cohort ^1^		Unadj		Age Std ^2^	Unadj		Age Std ^2^	
	n (total =524)	%	n (total =269)	%	%	n (total =269)	%	%	p
**Cancer site**									
*Liver cancer*	189	36%	97	36%		92	36%		
*Stomach cancer*	335	64%	172	64%		163	64%		
**Sex**									0.104
*Male*	349	67%	170	63%	64%	179	70%	70%	
*Female*	175	33%	99	37%	36%	76	30%	30%	
**Age** (years)									
*25-49*	105	20%	73	27%	-	32	13%	-	
*50-64*	152	29%	96	36%	-	56	22%	-	
*65-74*	139	27%	64	24%	-	75	29%	-	
*75+*	128	24%	36	13%	-	92	36%	-	
*Mean age (SD)*	64 (15)		60 (14)			68 (14)			**<0.001**
**Stage** (TNM)									0.688
*I*	84	16%	41	15%	16%	43	17%	17%	
*II*	93	18%	51	19%	19%	42	16%	17%	
*III*	116	22%	59	22%	22%	57	22%	22%	
*IV*	226	43%	118	44%	42%	108	42%	43%	
*Unstaged*	5	1%	0	-	-	5	2%	2%	
**NZDep** (Deciles)									**<0.001**
*Lowest deprivation: 1-2*	38	7%	12	5%	5%	26	10%	10%	
*3-4*	61	12%	18	7%	7%	43	17%	17%	
*5-6*	81	16%	32	12%	11%	49	20%	19%	
*7-8*	112	22%	47	18%	18%	65	26%	27%	
*Highest deprivation: 9-10*	217	43%	151	58%	60%	66	27%	27%	
**Rurality**									**<0.001**
*Urban*	371	73%	166	64%	65%	205	82%	83%	
*Independent urban*	77	15%	51	20%	19%	26	10%	10%	
*Rural*	61	12%	43	17%	16%	18	7%	7%	
**Common comorbidities**									
*Angina*	78	16%	36	13%	17%	42	16%	14%	0.376
*Hypertension*	200	39%	106	39%	46%	94	37%	33%	**<0.001**
*Myocardial infarction*	41	9%	16	6%	7%	25	10%	8%	0.735
*Arrhythmia*	84	17%	38	14%	18%	46	18%	16%	0.545
*CHF*	51	9%	29	11%	15%	22	9%	7%	**<0.001**
*Mild CPD*	32	6%	17	6%	7%	15	6%	5%	0.385
*Mod/Severe CPD*	41	9%	16	6%	8%	25	10%	8%	0.818
*CVD*	53	12%	19	7%	9%	34	13%	11%	0.418
*Uncomplicated diabetes*	131	23%	74	28%	29%	57	22%	22%	0.100
*Other primary tumour*	48	10%	19	7%	8%	29	11%	10%	0.595
*Mod/severe renal disease*	27	4%	19	7%	9%	8	3%	3%	**<0.001**
*Obesity*	45	7%	31	12%	10%	14	5%	5%	**0.028**
**C3 Index category** ^**3**^									**0.050**
*0*	212	40%	108	40%	36%	104	41%	43%	
*1*	92	18%	47	17%	17%	45	18%	19%	
*2*	74	14%	35	13%	13%	39	15%	15%	
*3*	146	28%	79	29%	34%	67	26%	23%	

^1^Total cohort results weighted to total liver/stomach cancer population (2006–2008). ^2^Age standardised to total NZ cancer population (2006–2008). ^3^ C3 index category: ’0’ (C3 Index score < =0), ‘1’ (0 < score < =1), ‘2’ (1 < score < =2) and ‘3’ (score >2).

**Table 2:** **Crude all-cause and cancer-specific 2-year survival by cancer site, including median survival time in months**

		All-cause survival		Cancer-specific survival	
	n (total)	% surviving at 2 years	Median (months)	% surviving at 2 years	Median (months)
***Combined***					
*Total*	524	27%	8.4	29%	8.9
*Māori*	269	26%	8.4	28%	8.6
*Non-Māori*	255	29%	8.8	31%	9.0
***Liver***					
*Total*	189	29%	6.6	30%	7.5
*Māori*	97	26%	6.6	27%	7.5
*Non-Māori*	92	32%	6.6	33%	8.0
***Stomach***					
*Total*	335	27%	9.0	29%	9.5
*Māori*	172	26%	8.9	28%	9.7
*Non-Māori*	163	27%	9.0	30%	9.3

The distribution of the cohort by comorbidity (‘C3 Index’) category is shown in Table 1 (and Additional file 1: Table S1), with the range being 0 to 12.8. Proportionally, the greatest number of patients were recorded as having no comorbidity (C3 Index category ‘0’; weighted proportion of total cohort: 40%), but 28% in the total cohort had levels of comorbidity in the highest category (C3 index category 3). Age-standardised results suggested that proportionally fewer Māori patients had no comorbidity (age-standardised proportion: Māori 36%, non-Māori 43% in the total cohort) and proportionally more had a comorbidity score >2 (Māori: 34%, non-Māori 23%). Linear regression analysis confirmed that Māori tended to have a higher comorbidity burden than non-Māori (age adjusted mean difference: 0.42, 95% CI 0.06-0.78). Additional file 1: Table S2 provides the other characteristics of the cohort (site, gender, age, stage, deprivation and rurality) by comorbidity category.

Hypertension was the most common comorbid condition identified from the clinical notes review data (weighted proportion of total cohort: 39%), with Māori more likely to suffer this condition than non-Māori (age-standardised proportions: Māori: 46%, non-Māori 33%; p <0.01). Uncomplicated diabetes (23%), arrhythmia (17%) and angina (16%) were also common. Māori were more likely to be reported as having congestive heart failure (age-standardised proportion: Māori: 15%, non-Māori 7%, p <0.01), moderate/severe renal disease (Māori 9%, non-Māori: 3%; p <0.01) and obesity (Māori: 10%, non-Māori: 5%; p =0.03) than non-Maori.

The likelihood of receiving curative treatment reduced in a linear manner with increasing comorbidity (Table 3). For example, those with a C3 Index score of 6 had substantially reduced odds of receipt of curative surgery compared to a C3 Index score of 0, even after adjusting for age, sex, site of cancer, stage of disease, ethnicity, deprivation and rurality (adjusted OR: 0.32, 95% CI 0.13-0.78).

**Table 3:** **Association between comorbidity and treatment receipt among stage I-III patients: unadjusted and adjusted odds ratios**

Impact of comorbidity evaluated at C3 score of:*	Unadjusted OR (95% CI)	Adj OR ** (95% CI)
0	1.00	1.00
1	0.70 (0.50-0.98)	0.92 (0.59-1.42)
2	0.53 (0.31-0.89)	0.80 (0.40-1.6)
3	0.42 (0.23-0.78)	0.66 (0.29-1.49)
6	0.29 (0.15-0.56)	0.32 (0.13-0.78)

* evaluation of the OR at a score of *1, 2, 3* and *6* (in relation to a score of *0*). No results are presented beyond 6 as the 95^th^ percentile of the C3 Index score distribution was 6.74, which means estimates of the OR beyond this point on the scale are of limited utility.

** adjusted for age (continuous), sex, site (stomach/ liver), stage (I, II, III), deprivation (continuous, in deciles), rurality (rural/other), ethnicity (Māori/ Non-Māori).

Receipt of curative surgery was strongly associated with mortality. Those Stage I-III patients who did not receive surgery were three and a half times more likely to die than those who received surgery (cancer-specific HR =3.68, 95% CI 2.49-5.45; all-cause HR =3.54, 95% CI 2.33-5.34; both models adjusted for age, sex, site, stage, ethnicity, deprivation, rurality and comorbidity).

There was an association between comorbidity and cancer survival but this was non-linear among those with stage I-III disease after adjusting for age, sex, site, stage, ethnicity, deprivation and rurality (Table 4). For example, while a C3 Index score of 3 was associated with a 54-62% increased likelihood of death compared to those with a score of 0 (adjusted HRs: cancer-specific 1.62, 95% CI 1.03-2.54; all-cause 1.54, 95% CI 1.01-2.35), there tended to be a drop-off in risk of mortality beyond this point (e.g. C3 index score 6 vs 0, cancer-specific HR: 1.15, 95% CI 0.71-1.87; all-cause HR: 1.44, 95% CI 0.93-2.23).

**Table 4:** **Cancer-specific and all-cause survival by category of comorbidity among those with Stage I-III disease**

	Cancer-specific mortality Hazard Ratios (95% CI)		
Impact of comorbidity evaluated at C3 Score of:*	Unadjusted	Model 1^1^	Model 2^2^
0	1.00	1.00	1.00
1	1.38 (1.11-1.71)	1.33 (1.04-1.71)	1.24 (0.96-1.6)
2	1.68 (1.2-2.36)	1.56 (1.05-2.31)	1.36 (0.91-2.04)
3	1.86 (1.26-2.74)	1.62 (1.03-2.54)	1.34 (0.84-2.13)
6	1.68 (1.08-2.61)	1.15 (0.71-1.87)	0.85 (0.51-1.4)
	**All-cause mortality Hazard Ratios (95% CI)**		
C3 score*	Unadjusted	Model 1^1^	Model 2^2^
0	1.00	1.00	1.00
1	1.30 (1.06-1.6)	1.25 (0.99-1.58)	1.17 (0.92-1.48)
2	1.57 (1.14-2.17)	1.43 (0.99-2.07)	1.27 (0.87-1.85)
3	1.77 (1.23-2.56)	1.54 (1.01-2.35)	1.28 (0.83-1.99)
6	1.97 (1.33-2.9)	1.44 (0.93-2.23)	1.04 (0.66-1.63)

^1^Adjusted for age, sex, site, stage, ethnicity, deprivation and rurality ^2^ Adjusted for all previous variables plus receipt of treatment. * evaluation of the OR at a score of *1, 2, 3* and *6* (in relation to a score of *0*). No results are presented beyond 6 as the 95^th^ percentile of the C3 Index score distribution was 6.74, which means estimates of the OR beyond this point on the scale are of limited utility.

The introduction of receipt of curative surgery into the models substantially reduced excess mortality among those with comorbidity; for example, the adjusted excess mortality among those with a C3 Index score of 3 (compared to 0) was reduced by almost half, once treatment receipt was included in the model (reduction in excess mortality: cancer-specific 45%; all-cause 48%; treatment-adjusted HRs: cancer-specific 1.34, 95% CI 0.84-2.13; all-cause HR 1.28, 95% CI 0.83-1.99;).

On examining ethnic differences in treatment receipt and survival (Table 5), Māori and non-Māori patients had similar odds of curative surgery after adjusting for age, sex, site, stage and comorbidity (adjusted odds ratio [OR]: non-Māori referent; Māori 1.07, 95% CI 0.57-1.99). Māori appeared more likely to die than non-Māori after adjusting for age, sex, site, stage of disease, comorbidity and receipt of treatment, with an estimated 33-36% higher mortality; although confidence intervals around these estimates included the null (adjusted HRs: cancer-specific 1.36, 95% CI 0.97-1.90; all-cause 1.33, 95% CI 0.97-1.82). Additionally adjusting for access to healthcare factors (deprivation and rurality) accounted for 25% of the remaining excess cancer-specific and 36% of the excess all-cause mortality.

**Table 5:** **Comparison of Māori compared with non-Māori patients (Stage I-III): receipt of definitive treatment and excess mortality**

	Receipt of definitive treatment	Cancer-specific	All-cause
	OR (95% CI)	HR (95% CI)	HR (95% CI)
Crude	1.26 (0.79-2.02)	1.03 (0.76-1.4)	1.01 (0.76-1.35)
+ age, sex and site	0.9 (0.51-1.61)	1.28 (0.92-1.76)	1.25 (0.92-1.70)
+ stage	0.9 (0.49-1.63)	1.33 (0.96-1.85)	1.29 (0.95-1.76)
+ comorbidity*	1.07 (0.57-1.99)	1.23 (0.88-1.72)	1.23 (0.89-1.69)
+ treatment receipt	-	1.36 (0.97-1.90)	1.33 (0.97-1.82)
+ health service access factors**	1.12 (0.54-2.35)	1.27 (0.88-1.85)	1.21 (0.85-1.72)

*Adjusted for both comorbidity count and C3 index.

**Adjusted for deprivation (NZDep decile) and rurality.

## Discussion

In a cohort of patients with liver and stomach cancers, increasing levels of comorbidity were associated with a reducing likelihood of receipt of definitive treatment. Receipt of definitive treatment was, in turn, strongly associated with survival. Comorbidity was also associated with poorer cancer-specific and all-cause survival, although the association levelled off for those with the highest comorbidity scores. Receipt of curative surgery substantially reduced excess mortality among those with comorbidity, the extent of which varied non-linearly by level of comorbidity. Māori patients were about a third more likely to die from their cancer or all-causes, after adjusting for age, sex, site and stage of disease, but this was largely not explained by comorbidity or receipt of definitive treatment. Access to healthcare factors accounted for a quarter of cancer-specific and a third of all-cause survival difference.

The high prevalence of comorbidity among this group of patients with stomach and liver cancers was expected given the risk profile of these cancers which includes smoking, alcohol and obesity as well as chronic infection [43]. Research has clearly established that cancer patients who also have other chronic conditions are less likely to receive definitive treatment for their cancer, although evidence relating to liver and stomach cancers specifically is sparse [6–14]. Vignette-based studies that ask clinicians to consider decisions on the basis of summarised information about hypothetical patients have also consistently found that surgeons and oncologists are less likely to refer or recommend treatment for cancer patients with comorbidity [44–46].

Intuitively, it is not unreasonable for clinicians (and their patients) to be concerned about the potential for higher risk of complications or toxicity from cancer treatment among those with comorbidity. However, the evidence among cancer patients relating to the risk of complications from treatment among those with comorbidity is conflicting. While some studies suggest that those with comorbidity are at greater risk of complications [11, 47, 48], others have reported that there is no or minimal difference in rates of complications between those with and without comorbidity [49–55]. This suggests that the large differences in treatment offer and receipt between these groups with and without comorbidity may not always be justifiable from the point of view of treatment toxicity and complications. Furthermore, as demonstrated by this and other studies, those with comorbidity who receive definitive treatment appear to have improved chance of survival [13, 49, 56]. However, the question of the extent to which treatment (or lack thereof) impacts on survival for those with comorbidity ideally requires randomised controlled trial evidence. Such trials frequently exclude older patients and those with comorbidity are, such that the evidence produced relates to interventions that apply to younger, healthier patients [57–61]. Given the prevalence of comorbidity among cancer patients, it would seem this well-recognised issue needs to be addressed.

Our finding that Māori patients had poorer survival than non-Māori patients is consistent with research relating to other cancers [20, 62–65]. Māori patients differed from non-Māori patients in a number of respects; they were younger at diagnosis (mean age 60 vs. 68 years), had somewhat higher levels of comorbidity (34% vs 23% in highest category) and were substantially more likely to live in more deprived (60% vs 27%) and rural areas (16% vs 7%). However, contrary to our expectations, there were no differences between Māori and non-Māori patients in terms of stage at diagnosis or receipt of curative surgery. Comorbidity and treatment receipt were not able to explain the one-third survival difference between Māori and non-Māori patients, although access to health care factors (deprivation and rurality) accounted for some of this disparity. It may be that these variables reflect some aspects of the timeliness or quality of treatment received that was more subtle than could be assessed in this study.

There are some potential limitations of our study. First, it was an observational study, and the decision to offer treatment is likely to be related to a range of variables for which we did not have information – including both patient factors (such as social support) and disease factors (such as tumour size). As such, the receipt (or non-receipt) of curative surgery is almost certainly a proxy marker for other prognostic indicators. Since these other unmeasured variables may also be related to comorbidity, the observed reduction in excess mortality among those with high comorbidity following the addition of curative surgery receipt to our survival models may be an overestimate. Put another way, those who are selected to receive surgery, even after adjusting for comorbidity, may be those who have a better prognosis and/or are healthier than those who are not selected for surgery. Furthermore, any global measure of comorbidity is, by necessity, a simplification of reality. We were unable to assess whether there were certain comorbid conditions that were more important than others in terms of receipt of treatment or poor outcomes due to the sample size. Because nearly half the patients in this study had stage IV disease at diagnosis, and because both stomach and liver cancers tend to have poor prognosis for all groups of patients, our ability to discriminate between groups of patients may have been limited. This means there was a lack of precision around some of our estimates and resulting wide confidence intervals.

This study has several significant strengths; first, we were able to secure complete TNM staging data for 99% of our cohort. Second, we collected data on the total eligible Māori stomach and liver cancer population (and an equal-number of randomly-selected non-Māori), meaning that we had equal explanatory power for both Māori and non-Māori patients. Third, we used two measures of comorbidity; one based on data from clinical notes and the other specifically designed and validated for use among cancer populations (liver and stomach cancers included) [38]. This approach is likely to have reduced the mismeasurement of the complex construct of patient comorbidity compared with other more general approaches.

## Conclusions

We observed that patients with high comorbidity were a) substantially less likely to receive curative surgery and b) more likely to die than those without comorbidity. Accounting for provision of curative treatment to those with high comorbidity significantly reduced this excess mortality. There was some evidence of an increased comorbidity burden among indigenous Māori patients, but no evidence of an inequality in receipt of curative surgery. Despite adjusting for age, gender, stage of disease, comorbidity and receipt of curative surgery, indigenous Māori patients were still one-third more likely to die of cancer-specific or all-causes than non-Māori.

## Author information

DS; MBChB, MPH, PhD. Associate Professor. Public health physician and Director of Cancer Control and Screening Research Unit. JG; PhD. Research Fellow, Cancer Control and Screening Research Unit. JS; PhD. Biostatistician, Senior Research Fellow, Biostatistical Group. JK; MBChB, MD. Associate Professor. Upper gastrointestinal surgeon.

## Electronic supplementary material

Additional file 1: **Figure S1.** Direct Acyclic Graphs (DAGS) for key relationships between comorbidity, receipt of treatment, ethnicity and survival among patients with stomach and liver disease. **Table S1.** Characteristics of the study population (excluding Stage 4 patients): proportions by site sex, age, stage, deprivation, rurality and comorbidity. **Table S2.** Characteristics of the study population by C3 index category*: proportions by site, gender, age, stage, deprivation and rurality. (DOCX 76 KB)
