# Supplementary material for: A retrospective cohort study of patients with stomach and liver cancers: the impact of comorbidity and ethnicity on cancer care and outcomes
Source: BMC Cancer. 2014 Nov 7;14:821. doi: 10.1186/1471-2407-14-821 (PMC4233029; doi:10.1186/1471-2407-14-821)
Supplement: Supplementary file 1 — Additional file 1: Figure S1. Direct Acyclic Graphs (DAGS) for key relationships between comorbidity, receipt of treatment, ethnicity and survival among patients with stomach and liver disease. Table S1. Characteristics of the study population (excluding Stage 4 patients): proportions by site sex, age, stage, deprivation, rurality and comorbidity. Table S2. Characteristics of the study population by C3 index category*: proportions by site, gender, age, stage, deprivation and rurality. (DOCX 76 KB) [file 12885_2014_5000_MOESM1_ESM.docx]

**Additional file**

**Figure S1:** Direct Acyclic Graphs (DAGS) for key relationships between comorbidity, receipt of treatment, ethnicity and survival among patients with stomach and liver disease.


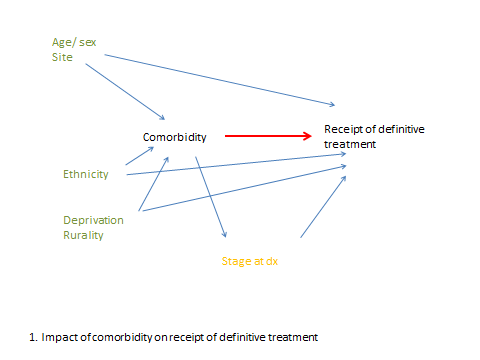

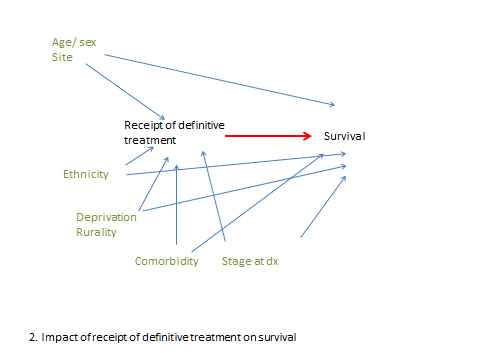

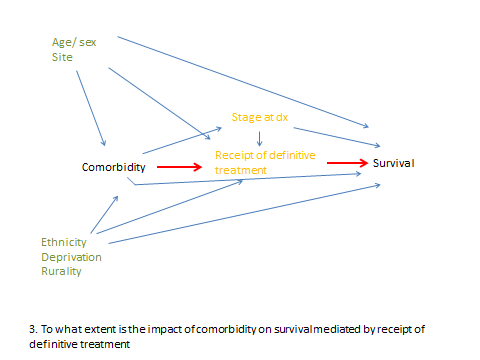

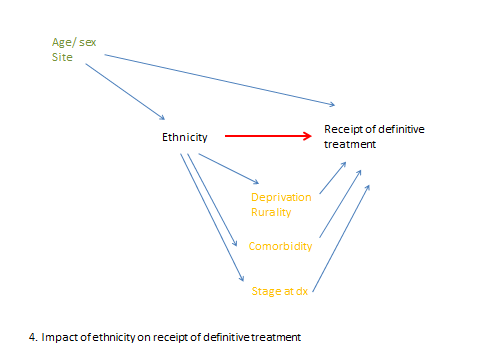


**Table S1:** Characteristics of the study population (excluding Stage 4 patients): proportions by site sex, age, stage, deprivation, rurality and comorbidity.

|  |  | | **Māori** | | | **Non-Māori** | | |  |
| --- | --- | --- | --- | --- | --- | --- | --- | --- | --- |
|  | **Total cohort^1^** | | **Unadj** | | **Age Std** | **Unadj** | | **Age Std** |  |
|  | *n (total =298)* | *%* | *n (total =151)* | *%* | *%* | *n (total =147)* | *%* | *%* | *p* |
| **Cohort** |  |  |  |  |  |  |  |  |  |
| *Liver Cancer* | 121 | 41% | 64 | 42% |  | 57 | 39% |  |  |
| *Stomach Cancer* | 177 | 59% | 87 | 58% |  | 90 | 61% |  |  |
| **Gender** |  |  |  |  |  |  |  |  | 0.027 |
| *Male* | 201 | 67% | 94 | 62% | 62% | 107 | 73% | 74% |  |
| *Female* | 97 | 33% | 57 | 38% | 38% | 40 | 27% | 26% |  |
| **Age** (years) |  |  |  |  |  |  |  |  |  |
| *25-49* | 56 | 19% | 39 | 26% | - | 17 | 12% | - |  |
| *50-64* | 88 | 30% | 57 | 38% | - | 31 | 21% | - |  |
| *65-74* | 77 | 26% | 30 | 20% | - | 47 | 32% | - |  |
| *75+* | 77 | 26% | 25 | 17% | - | 52 | 35% | - |  |
| *Mean Age (SD)* | 65 (15) |  | 60 (14) |  |  | 69 (14) |  |  | **<0.001** |
| **Stage** (TNM) |  |  |  |  |  |  |  |  | 0.413 |
| *I* | 84 | 28% | 41 | 27% | 28% | 42 | 29% | 29% |  |
| *II* | 93 | 31% | 51 | 34% | 34% | 57 | 29% | 30% |  |
| *III* | 116 | 39% | 59 | 39% | 39% | 5 | 39% | 38% |  |
| *Unstaged* | 5 | 2% | 0 | 0% | - | 6 | 3% | 3% |  |
| **NZDep** (Deciles) |  |  |  |  |  |  |  |  | **<0.001** |
| *Lowest Deprivation: 1-2* | 21 | 7% | 6 | 4% | 4% | 15 | 10% | 10% |  |
| *3-4* | 39 | 13% | 10 | 7% | 6% | 29 | 20% | 21% |  |
| *5-6* | 46 | 16% | 14 | 10% | 9% | 32 | 22% | 21% |  |
| *7-8* | 57 | 20% | 28 | 19% | 20% | 29 | 20% | 20% |  |
| *Highest Deprivation: 9-10* | 127 | 44% | 87 | 60% | 61% | 40 | 28% | 27% |  |
| **Rurality** |  |  |  |  |  |  |  |  | **<0.001** |
| *Urban* | 217 | 75% | 24 | 65% | 62% | 12 | 85% | 84% |  |
| *Independent Urban* | 36 | 12% | 27 | 17% | 18% | 10 | 8% | 8% |  |
| *Rural* | 37 | 13% | 123 | 19% | 19% | 0 | 7% | 7% |  |
| **Common Comorbidities** |  |  |  |  |  |  |  |  |  |
| *Angina* | 52 | 19% | 24 | 16% | 20% | 28 | 19% | 16% | 0.366 |
| *Hypertension* | 126 | 44% | 64 | 42% | 49% | 62 | 42% | 38% | **0.043** |
| *Myocardial Infarction* | 23 | 9% | 7 | 5% | 6% | 16 | 11% | 9% | 0.501 |
| *Arrythmia* | 50 | 17% | 24 | 16% | 21% | 26 | 18% | 16% | 0.312 |
| *CHF* | 35 | 10% | 21 | 14% | 19% | 14 | 10% | 8% | **<0.001** |
| *Mild CPD* | 20 | 6% | 12 | 8% | 8% | 8 | 5% | 4% | 0.195 |
| *Mod/Severe CPD* | 28 | 11% | 12 | 8% | 9% | 16 | 11% | 10% | 0.891 |
| *CVD* | 31 | 13% | 10 | 7% | 8% | 21 | 14% | 12% | 0.292 |
| *Uncomplicated Diabetes* | 86 | 27% | 44 | 29% | 31% | 42 | 29% | 27% | 0.588 |
| *Other Primary Tumour* | 34 | 13% | 11 | 7% | 9% | 23 | 16% | 13% | 0.327 |
| *Mod/Severe Renal Disease* | 15 | 5% | 9 | 6% | 7% | 6 | 4% | 4% | **0.315** |
| *Obesity* | 25 | 6% | 19 | 13% | 11% | 6 | 4% | 3% | **0.013** |
| **C3 Index Category** |  |  |  |  |  |  |  |  | **0.511** |
| *0* | 118 | 40% | 62 | 41% | 36% | 56 | 38% | 41% |  |
| *1* | 53 | 18% | 26 | 17% | 17% | 27 | 18% | 20% |  |
| *2* | 36 | 12% | 18 | 12% | 12% | 18 | 12% | 12% |  |
| *3* | 91 | 31% | 45 | 30% | 34% | 46 | 31% | 27% |  |

**Table S2:** Characteristics of the study population by C3 index category*: proportions by site, gender, age, stage, deprivation and rurality

|  | **C3 Index Category** | | | | | | | | | | | | |
| --- | --- | --- | --- | --- | --- | --- | --- | --- | --- | --- | --- | --- | --- |
|  | **0** | | | **1** | | | **2** | | | **3+** | | |  |
|  |  | **Crude** | **Age Std** |  | **Crude** | **Age Std** |  | **Crude** | **Age Std** |  | **Crude** | **Age Std** |  |
|  | *n (total=* | *%* | *%* | *n (total=* | *%* | *%* | *n (total=* | *%* | *%* | *n (total=* | *%* | *%* | *P* |
|  | *212)* |  |  | *92)* |  |  | *74)* |  |  | *146)* |  |  |  |
| **Cohort** |  |  |  |  |  |  |  |  |  |  |  |  |  |
| *Liver Cancer* | 53 | 25% |  | 50 | 54% |  | 31 | 42% |  | 55 | 38% |  |  |
| *Stomach Cancer* | 159 | 75% |  | 42 | 46% |  | 43 | 58% |  | 91 | 62% |  |  |
| **Gender** |  |  |  |  |  |  |  |  |  |  |  |  | **0.045** |
| *Male* | 132 | 62% | 65% | 66 | 72% | 69% | 58 | 78% | 79% | 93 | 64% | 65% |  |
| *Female* | 80 | 38% | 35% | 26 | 28% | 31% | 16 | 22% | 21% | 53 | 36% | 35% |  |
| **Age** (years) |  |  |  |  |  |  |  |  |  |  |  |  |  |
| *25-49* | 61 | 29% | - | 26 | 28% | - | 11 | 15% | - | 7 | 5% | - |  |
| *50-64* | 63 | 30% | - | 31 | 34% | - | 24 | 32% | - | 34 | 23% | - |  |
| *65-74* | 46 | 22% | - | 18 | 20% | - | 20 | 27% | - | 55 | 38% | - |  |
| *75+* | 42 | 20% | - | 17 | 18% | - | 19 | 26% | - | 50 | 34% | - |  |
| *Mean Age (SD)* | 60 (15) |  |  | 60 (14) |  |  | 66 (13) |  |  | 71 (11) |  |  | **-** |
| **Stage** (TNM) |  |  |  |  |  |  |  |  |  |  |  |  | 0.711 |
| *I* | 36 | 17% | 17% | 15 | 16% | 17% | 7 | 9% | 9% | 26 | 18% | 15% |  |
| *II* | 36 | 17% | 17% | 14 | 15% | 16% | 13 | 18% | 17% | 30 | 21% | 25% |  |
| *III* | 44 | 21% | 20% | 24 | 26% | 27% | 16 | 22% | 23% | 32 | 22% | 20% |  |
| *IV* | 94 | 44% | 45% | 39 | 42% | 40% | 38 | 51% | 52% | 55 | 38% | 38% |  |
| *Unstaged* | 2 | 1% | 1% | 0 | 0% | - | 0 | 0% | - | 3 | 2% | 2% |  |
| **NZDep** (Quintile) |  |  |  |  |  |  |  |  |  |  |  |  | 0.478 |
| *1-2* | 17 | 8% | 9% | 7 | 8% | 8% | 6 | 8% | 8% | 8 | 6% | 4% |  |
| *3-4* | 23 | 11% | 12% | 10 | 11% | 11% | 7 | 10% | 10% | 21 | 14% | 11% |  |
| *5-6* | 28 | 14% | 15% | 20 | 22% | 22% | 10 | 14% | 14% | 23 | 16% | 13% |  |
| *7-8* | 41 | 20% | 21% | 19 | 21% | 21% | 23 | 32% | 32% | 29 | 20% | 24% |  |
| *9-10* | 92 | 46% | 44% | 34 | 38% | 38% | 27 | 37% | 35% | 64 | 44% | 47% |  |
| **Rurality** |  |  |  |  |  |  |  |  |  |  |  |  | 0.315 |
| *Urban* | 139 | 69% | 68% | 67 | 74% | 77% | 55 | 75% | 74% | 110 | 76% | 78% |  |
| *Independent Urban* | 30 | 15% | 15% | 15 | 17% | 15% | 13 | 18% | 18% | 19 | 13% | 13% |  |
| *Rural* | 32 | 16% | 17% | 8 | 9% | 8% | 5 | 7% | 8% | 16 | 11% | 9% |  |
